# Acute suppression of lower limb spasm by sacral afferent stimulation for people with spinal cord injury: A pilot study

**DOI:** 10.1017/wtc.2024.4

**Published:** 2024-04-05

**Authors:** Sarah Massey, Sean Doherty, Lynsey Duffell, Mike Craggs, Sarah Knight

**Affiliations:** 1 Aspire Centre for Rehabilitation Engineering and Assistive Techonologies, University College London, London, UK; 2 Department of Medical Physics and Biomedical Engineering, University College London, London, UK; 3 London Spinal Cord Injury Centre, Royal National Orthopaedic Hospital, London, UK

**Keywords:** spinal cord injury, spasticity, neuromodulation, sacral afferent stimulation

## Abstract

Lower limb spasm and spasticity may develop following spinal cord injury (SCI), causing hyper-excitability and increased tone, which can impact function and quality of life. Pharmaceutical interventions for spasticity may cause unwanted side effects such as drowsiness and weakness. Invasive and non-invasive electrical stimulation has been shown to reduce spasticity without these side effects. The aim of this study was to investigate the effect of sacral afferent stimulation (SAS), through surface electrical stimulation of the dorsal genital nerve (*N* = 7), and through implanted electrodes on the sacral afferent nerve roots, on lower limb spasm and spasticity (*N* = 2). Provoked spasms were interrupted with conditional SAS, where stimulation commenced following a provoked spasm, or unconditional stimulation, which was applied continuously. Conditionally and unconditionally applied SAS was shown to suppress acute provoked spasms in people with SCI. There was a statistically significant reduction in area under the curve of quadriceps electromyography during acute spasm with SAS compared to a control spasm. These results show that SAS may provide a safe, low-cost method of reducing acute spasm and spasticity in people living with SCI. SAS through implanted electrodes may also provide an additional function to sacral nerve stimulation devices.

## 1. Introduction

Involuntary spasms of the lower limbs are a common manifestation of the spasticity associated with spinal cord injury (SCI). Spasticity can be attributed to the isolation of spinal reflex arcs from inhibitory mechanisms following SCI, which allows spasm (involuntary muscle contraction) to occur more easily (Biering-Sørensen et al., 2006; Lapeyre et al., 2010). Acute involuntary spasms are often initiated during maneuvres such as wheelchair transfers, or other noxious stimuli such as full bladder or rectum. Around 65% of people have spasticity at discharge following traumatic SCI (Holtz et al., 2017), increasing to 78% for chronic SCI (Maynard et al., 1990).

The most common mode of treatment is the use of pharmaceutical agents including baclofen, which interferes with the neuromuscular transmission (Lapeyre et al., 2010). Although these drugs are quite effective at reducing symptoms of spasticity (joint stiffness and increased muscle tone), they have less effect on acute spasms and are associated with side effects such as drowsiness and blurred vision (Royal College of Physicians, 2018). Injections of botulinum toxin can be beneficial if spasticity is localised, but cannot be used for generalised spasticity. Some people also find that spasticity has some positive benefits such as maintaining muscle bulk and are therefore reluctant to reduce tone completely.

Neuromodulation is a technique that restores function through modification of the residual, or altered neurological system (Minassian et al., 2012) using electrical stimulation targeting either the peripheral or central nervous system. Techniques include transcutaneous electrical nerve stimulation (TENS), functional electrical stimulation (FES), epidural (eSCS) or transcutaneous spinal cord stimulation (tSCS), and sacral nerve stimulation. Studies have shown that surface electrical stimulation of the hip adductors (Franek et al., 1988), lumbar dermatomes, (Bajd et al., 1985) and eSCS (Barolat et al., 1995) are capable of reducing lower limb spasticity following SCI. Reviews have reported beneficial effects of FES gait and cycling on spasticity (Bekhet et al., 2019; Luo et al., 2020; Massey et al., 2022). In addition, recent studies of eSCS and tSCS to promote restoration of locomotor function have also shown promising results in reducing spasticity (Hofstoetter et al., 2014, 2020; Angeli et al., 2018).

Sacral nerve neuromodulation has been used extensively to control bladder over-activity (a form of spasticity) in both neurogenic and non-neurogenic patients (Occhino and Siegel, 2010; Bartley et al., 2013; Sukhu et al., 2016; Vargas Luna et al., 2016). Stimulation of sacral afferents can be achieved both non-invasively, through surface electrical stimulation of the dorsal genital nerve (DGN), and invasively, with electrodes placed on the extradural sacral nerve roots (Medtronic Interstim). Kirkham et al. (2002) describe a modification of the Finetech-Brindley Sacral Anterior Root Stimulator (SARS) (Finetech Medical Limited, UK) implant, which is normally associated with a sacral deafferentation (SDAF), in which the sacral afferents are spared. This allows bladder emptying through electrical stimulation of the anterior sacral roots as normal, but enables electrical stimulation of the sacral afferents to neuromodulate over-activity of the bladder in SCI. Only one previous study reports the effect of sacral afferent electrical stimulation (SAS) on acute lower limb spasms; Halstead et al. (1993) reported a diminution of spasms in patients who had received rectal probe electrostimulation for ejaculation. However, there are also reports of the use of penile vibratory stimulation to relieve lower limb spasms in SCI (Halstead et al., 1993; Læssøe et al., 2004; Alaca et al., 2005).

The aim of this pilot study was to investigate the effect of electrical stimulation of the sacral afferents, as an adjuvant therapy, for the control of lower limb spasticity and acute spasm secondary to SCI. SAS was delivered either through surface electrical stimulation of the DGN, or through electrodes implanted on the sacral afferent nerves. We hypothesise that SAS can suppress acute spasm of the lower limbs and reduce muscle stiffness in people with SCI.

## 2. Methods

### 2.1. Participants

Local ethics approval was obtained from the Royal National Orthopaedic Hospital, and participants gave informed consent prior to entering the study. The inclusion criteria for the study were: male; over 18 years of age; SCI over 6 months ago; has lower limb spasticity; does not have a pacemaker; no injury to the lower limbs; no knee replacement. Participants were divided into two groups: those without an implant and those with a Finetech-Brindley SARS without SDAF. Participants were not requested to stop taking anti-spasmodic medication.

### 2.2. Sacral afferent stimulation

SAS was applied using two different techniques; through surface electrodes and implanted electrodes.

In participants without an implant, the DGN was stimulated through two pre-gelled surface electrodes (Alpine Biomed, Fountain Valley, CA) placed on the dorsal side of the penis, with the anode distal to the cathode. Electrical stimulation was delivered through a constant current isolated stimulator (DS7A, Digitimer Ltd, UK).

An optimisation study was performed on an individual participant. Frequencies of 0, 5, 15, 25, 50, and 100 Hz, and pulse amplitudes of 0, 10, 20, 30, and 50 mA were tested while quadriceps electromyography (EMG) was measured. The area under the curve (AUC) of EMG responses was assessed to determine which parameters were most successful at reducing acute muscle spasm. Following this, a frequency of 15 Hz and pulse width of 200 μs were used for all participants. This optimisation study also concurred with the optimal stimulation frequency for neuromodulation of detrusor over-activity (Kirkham et al., 2001). Stimulation current was set to the highest tolerated current without provoking spasm.

For participants with a SPARS implant, stimulation was delivered directly to the afferent sacral roots via the implant. Stimulation was to select nerve roots at a frequency of 15 Hz, pulse width of 200 μs, and highest tolerated current.

Both techniques of SAS were applied either conditionally (commenced following a provoked spasm) or unconditionally (applied continuously), as described in previous literature (Kirkham et al., 2001; Doherty et al., 2019).

### 2.3. Assessment of spasticity and spasm

The Wartenburg Pendulum test (Bajd and Vodovnik, 1984) was used to assess lower limb spasticity. Electrogoniometers (Biometric Ltd, UK) were placed around the bilateral knee joints. Surface EMG electrodes (Biometric Ltd) were placed over the bilateral quadriceps (rectus femoris), with the ground electrode on the ankle joint. Electrogoniometry and EMG signals were sampled at 2 kHz and captured using Spike 2 software via an analog-to-digital converter (1401plus, Cambridge Electronic Design, UK).

**Figure 1.**
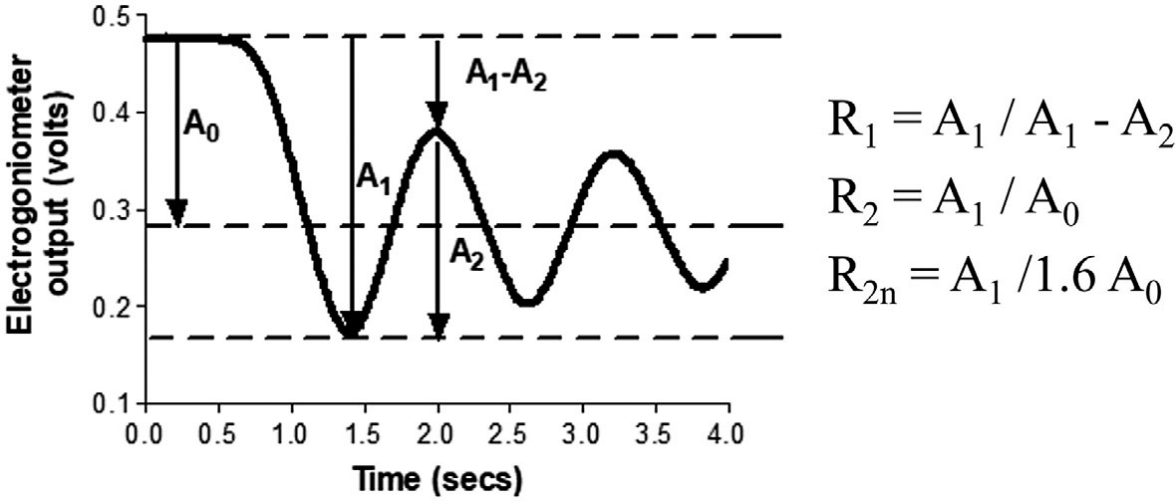
Calculation of Wartenberg parameters R1, R2, and R2n from oscillation of lower leg during pendulum test.

To assess spasticity, the participant sat in an upright position with their legs hanging over the end of the couch. Each leg was lifted to a horizontal position and allowed to fall under gravity. This was repeated three times for each leg. The R1, R2, and R2n values were calculated as described by Bajd and Vodovnik (1984) (see Figure 1). Pendulum tests were repeated with continuous SAS applied and without SAS.

### 2.4. Assessment of acute spasm

Acute lower limb spasms were provoked by rapidly changing position from supine to sitting. Quadriceps EMG were recorded and the area under the rectified and smoothed (0.05 s) curve was calculated (AUC). SAS was then applied conditionally or unconditionally.

### 2.5. Data and statistical analysis

All data were tested for normality using the Shapiro–Wilk’s test. Average R1, R2, and R2n values from three Wartenberg Pendulum tests of the dominant leg (assumed to be the same as the dominant hand) were calculated for control swings and with the application of SAS. The results were compared using a two-tailed paired *t*-test.

The effect of conditional and unconditional SAS on acute spasm was measured by comparing the AUC of the quadriceps EMG of the dominant leg with control spasms, using a Wilcoxon matched-paired signed rank test. All statistical analysis was carried out using GraphPad Prism 8.4.3 software (GraphPad Software Inc., Solana Beach, CA). Results were considered to be statistically significant for *p* < .05.

## 3. Results

Twelve male participants were recruited, of whom nine completed the study. Participant parameters are summarised in Table 1. Two participants had an SARS implant (without sacral deafferentation). Participant 8 had intrathecal electrodes in which the motor and sensory pathways of S3–4 were separated, allowing discrete stimulation of afferent and efferent nerves. Participant 9 had extradural electrodes whereby electrodes were placed around the mixed nerve. No adverse events occurred during this study.

**Table 1.** Participant characteristics

Participant No.	Age (years)	AIS	Time since injury (years)	Level	Injury	Cause of injury	Implant	Drugs
1	26	B	3	C6	Incomplete	Diving	None	Baclofen, Diazepam
2	23	A	4	T5/6	Complete	Motor vehicle	None	Baclofen
3	31	B	11	T4	Incomplete	Motor vehicle	None	None
4	41	B	7	C6	Incomplete	Motor vehicle	None	Baclofen
5	43	A	3	C6/7	Complete	Fall	None	None
6	23	A	4	T6	Incomplete	Motor vehicle	None	Dantrolene, Baclofen, Tizanidine
7	57	B	5	T5	Incomplete	Motor vehicle	None	Amitriptyline
8	38	A	7	T6	Complete	Motor vehicle	Intra–thecal	Baclofen
9	37	A	7	T4	Complete	Motor vehicle	Extra–dural	Baclofen, Dantrolene, Diazepam

### 3.1. Optimisation case study

**Figure 2.**
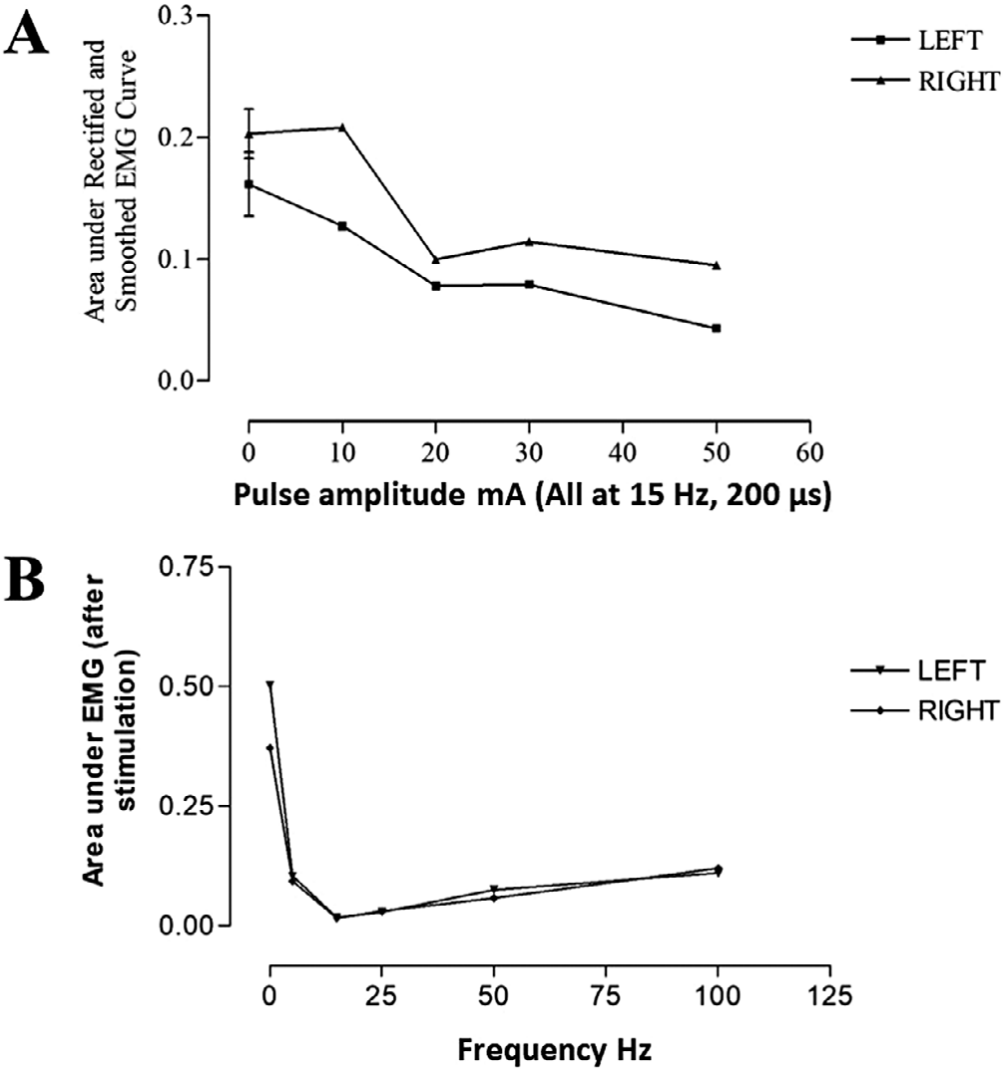
The effect of (a) pulse amplitude and (b) frequency on the area under EMG curve during dorsal genital nerve stimulation in one participant.

The dose-response curve for current amplitude and frequency of surface SAS on suppression of provoked lower limb spasm was investigated in one patient. The relationships between varying these parameters and AUC are shown in Figure 2. The optimal stimulation frequency in this participant was 15 Hz and the current intensity was 50 mA, which was their highest tolerated intensity. Following these results, the maximum tolerated intensity was used for remaining participants (40.7 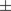 7.0 mA; mean 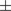 SD).

### 3.2. Effect of SAS on spasticity

**Figure 3.**
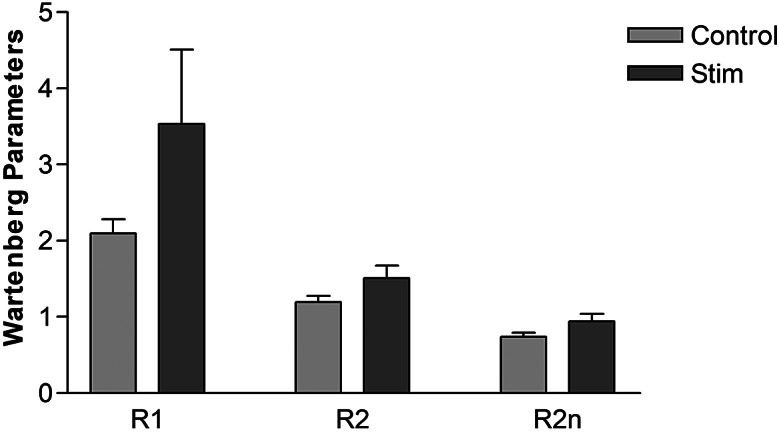
Knee joint spasticity measured using Wartenberg pendulum test for control swings and swing interrupted by sacral afferent stimulation. Error bars represent standard deviation. N = 4.

SAS delivered via the DGN reduced knee joint spasticity as demonstrated by trends of increase in Wartenberg parameters R1, R2, and R2n as shown in Figure 3. R1 increased from 2.1 ± 0.18 (mean ± SD) during control spasms to 3.5 ± 0.98 during SAS. Mean R2n for control swings was 0.7 ± 0.05 and 0.94 ± 0.1 during SAS, that is, approaching unity. Although there was a reduction in spasticity at the knee joint, demonstrated by an increase in R1 and R2n, these did not reach statistical significance.

### 3.3. Effect of SAS on acute spasm

**Figure 4.**
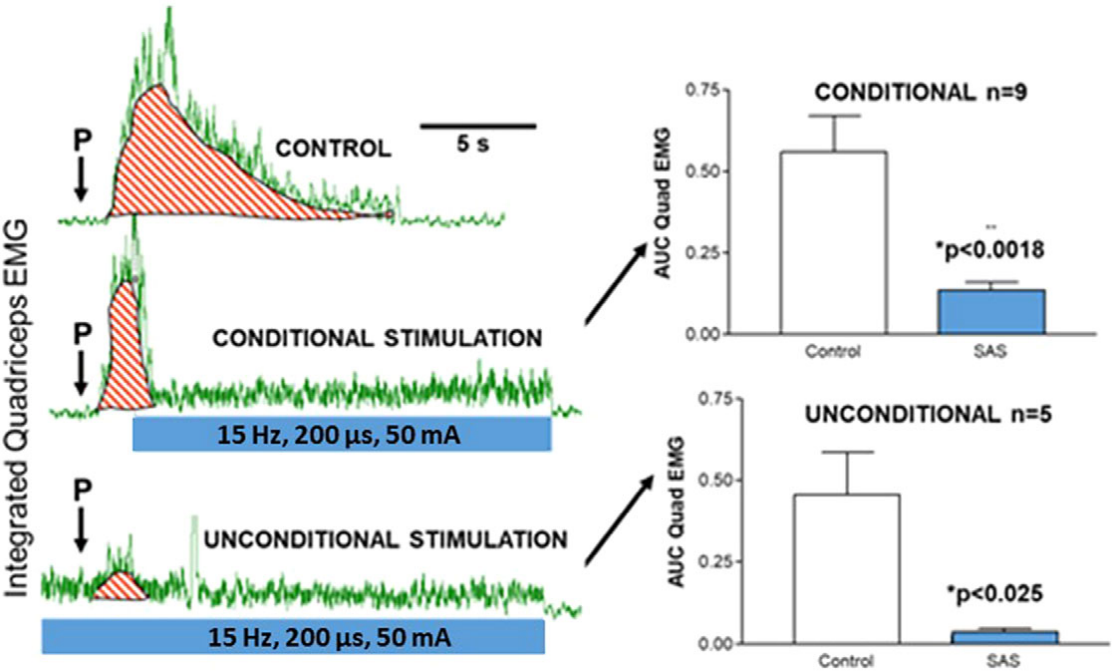
Left shows the provocation (P) of lower limb spasm on quadriceps EMG, and application of sacral nerve root neuromodulation, demonstrating control, conditional and unconditional stimulation for one participant. Red striped area represents area under the EMG trace. Right shows averaged area under the EMG curve of provoked spasm of optimised neuromodulation against control for conditional (*N* = 9) and unconditional stimulation (*N* = 5). Error bars represent standard deviation.

The effect of optimised conditional and unconditional SAS of the DGN on acute, provoked lower spasm is shown in Figure 4. Conditional SAS resulted in an immediate suppression of the spasm, seen as a reduction in AUC of the quadriceps signal compared to control spasms for the nine participants (*p* < 0.0018). When surface SAS was applied unconditionally, the provoked spasm that occurred was significantly reduced compared to the control (*p* < 0.025) in five participants.

**Figure 5.**
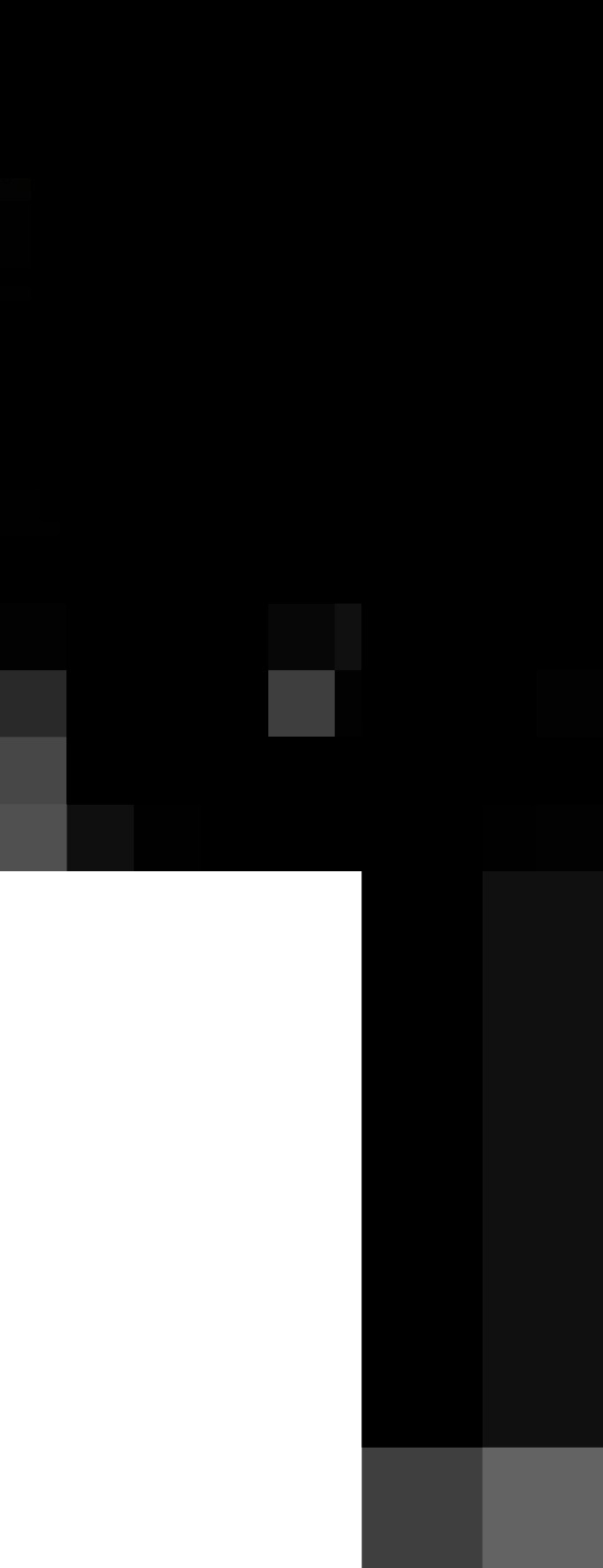
Effect of neuromodulation of sacral afferents through stimulation of posterior, sensory nerve roots via an implanted electrode (participant 8). The striped bar indicates when stimulation was being delivered.

The effect of SAS delivered through electrodes implanted on sacral afferent (posterior) nerve roots on lower limb spasm was also investigated. Figure 5 shows the effect of SAS on acute provoked spasm in Participant 8 with intrathecal electrodes. When SAS was turned on, there was an immediate suppression of provoked spasm as seen by reduction in quadriceps EMG activity and knee angle.

## 4. Discussion

This pilot study investigated the effects of neuromodulation of the sacral afferent nerves to control lower limb spasticity in people living with SCI. Our results showed that both non-invasive and invasive SAS may cause effective suppression of acutely provoked spasms in the lower limbs of nine males with SCI.

Surface stimulation of the DGN demonstrated increases in mean R1 and R2n which did not reach statistical significance, however, there were only four participants. The R1 for neurologically intact people has been found to be greater than 5, whereas in people with spasticity this is reduced to 2.6. In our study, the control value for R1 was 2.1 ± 0.18 which was increased to 3.5 ± 0.98 during SAS of the DGN.

In the current study, the R2n value was an average of 0.24 higher with DGN stimulation, compared to without. Other studies investigating non-invasive electrical stimulation for spasticity in SCI have not achieved this amount of reduction before and after at least 30 minutes of a single session of TENS (Bajd et al., 1985), tSCS (Hofstoetter et al., 2014), or FES cycling (Vargas Luna et al., 2016). Bajd et al. (1985) achieved the greatest change in mean R2n of 0.19; however, the six participants overall had a more severe R2n value at baseline. In contrast, another study achieved a change in mean R2n value from baseline following 60–100 minutes of FES cycling of 0.4 (Krause et al., 2008). Although the changes seen in the current study were lower than the study by Krause et al. (2008), our reduction in spasticity due to evoked spasms occurred immediately. Considering that there may be a larger anti-spastic effect during unconditional SAS, using this type of neuromodulation day-to-day would be relatively low-cost, easy to implement, and may reduce reliance upon pharmaceutical intervention.

Although TENS and FES can have an immediate effect on spasticity, long-term training using FES cycling may increase spasticity (Gant et al., 2018). This could be due to the muscle-strengthening effect of the stimulation or changes in the neurological signals. SAS does not increase muscle bulk and therefore would be unlikely to cause these side effects.

The suppressive effect was achieved through both SAS of the DGN and through direct stimulation of the sacral afferent nerve roots. In the participants with a SARS implant (without SDAF), suppression was optimal using the S34 nerve roots, however, suppression was also achieved through stimulation of the S2 nerve root. In the participant with separated motor and sensory nerve roots, the optimal suppression was achieved using the posterior or sensory nerve root. In addition, during stimulation of the motor pathways, the small movement of plantar flexors and toes was prevented. We can therefore infer that stimulation of the afferent pathway within the lumbosacral spinal cord was required to reduce the provoked spasm. It is thought that spinal cord stimulation is capable of modulating neurotransmitter release within the dorsal horn of the spinal cord (Parekh, 2017) and that improvements in spasticity are associated with a restoration of inhibitory pathways, mediating uncontrolled spinal reflexes following SCI (D’Amico et al., 2014). The neuromodulatory effect in this study was seen using both invasive and non-invasive SAS, suggesting that the mechanisms which reduced spasticity may have been activated via both peripheral and direct stimulation of the sacral afferents.

There were several limitations of this study. Firstly, the stimulation frequency and pulse width were selected based on results from one participant using the protocol presented in this study, as well as results in a previous study carried out by Kirkham et al. (2001), which aimed to suppress bladder overactivity. In the current study, SAS delivered in the range of 5–100 Hz was also investigated, however, this did not appear to have a significant effect on the suppression of acute spasm in one participant. A frequency of 15 Hz was used as this was compatible with that used for suppression of the overactive bladder. In addition, lower-frequency stimulation would be more beneficial in terms of battery life and implant power consumption. Another limitation of this study is that participants continued taking their anti-spasticity medication. All participants who took part were experiencing spasticity and spasm despite their medication; we were therefore investigating the use of SAS as an adjuvant therapy. Finally, the results of this study are based on small numbers of participants. Seven participants received SAS via the DGN and two via a SPARS implant. Although our results show a positive effect of SAS for suppression of acute spasm using both methods, a larger, randomised trial is required to confirm these results.

## 5. Conclusion

Both conditional and unconditional SAS could reduce quadriceps spasticity and spasm in this cohort of participants. These effects were present immediately following the application of SAS and were more pronounced following unconditional SAS. Results from this pilot study may present a potential safe, low-cost method of reducing acute lower limb spasticity in people living with SCI. This simple method may be implemented as an adjuvant therapy, both by people with and without a sacral root stimulation implant.

## Data Availability

Data can be made available to interested researchers upon request by email to the corresponding author.
